# Targeting Bruton tyrosine kinase with acalabrutinib attenuates murine sclerodermatous chronic graft versus host disease

**DOI:** 10.3389/fimmu.2026.1843217

**Published:** 2026-06-11

**Authors:** Vasantharaja Raguraman, Miranda Mysinger, Mohit Verma, Shanid Mohiyuddin, Nashwan Jabbour, Melissa Kesler, Trupti Joshi, Senthilnathan Palaniyandi, Gerhard C. Hildebrandt

**Affiliations:** 1 Division of Hematology and Medical Oncology, Department of Medicine, Ellis Fischel Cancer Center, University of Missouri, Columbia, MO, United States; 2 Division of Hematology & Blood and Marrow Transplantation, Department of Internal Medicine, Markey Cancer Center, University of Kentucky, Lexington, KY, United States; 3 Department of Biomedical Sciences, Joan C. Edwards School of Medicine, Marshall University, Huntington, WV, United States; 4 Department of Pathology and Laboratory Medicine, University of Kentucky, Lexington, KY, United States; 5 Department of Biomedical Informatics, Biostatistics and Medical Epidemiology, University of Missouri, Columbia, MO, United States

**Keywords:** Bruton tyrosine kinase, chronic graft-versus-host disease, fibrosis, inflammation, mast cells

## Abstract

**Background:**

Allogeneic hematopoietic stem cell transplantation (allo-HSCT) is considered the only curative option for certain malignant hematopoietic disorders, yet its benefits are limited by the development of graft-versus-host disease (GVHD). Chronic GVHD (cGVHD) is often characterized by chronic inflammation and fibrosis, propagated by alloreactive donor B- and T-cell involvement. Bruton tyrosine kinase (BTK) is the downstream effector in the B-cell receptor signaling pathway and further has a crucial role in mast cell activation, both of which are critical in the pathogenesis of cGVHD. Selective inhibition of BTK may be beneficial in ameliorating cGVHD pathogenesis. In this study, we evaluated the efficiency of acalabrutinib, a highly selective Bruton tyrosine kinase inhibitor, using a murine sclerodermatous cGVHD model.

**Methods:**

Recipient BALB/c mice received total body irradiation (800cGy), followed by infusion of bone marrow and splenocytes of either syngeneic (BALB/c) or allogeneic (B10.D2) mice, and were subsequently treated with acalabrutinib or control starting three weeks after HSCT until the end of week 8. Mice were monitored for clinical signs of GVHD, survival, organ pathology of skin and other organs, and inflammatory mediator expression in serum and skin. RNA sequencing and immunohistochemistry were performed in skin to understand the pharmacological mechanisms of BTK inhibition in sclerodermatous cGVHD.

**Results:**

Acalabrutinib treatment showed better survival, improved clinical cGVHD scores, and reduced skin pathology. Significant reductions in dermal thickness and skin fibrosis, and mast cell numbers in the acalabrutinib-treated group. *In vitro*, experiments on mast cell activation show that increased pro-inflammatory chemokines CCL2, CCL3, and CCL4 were effectively inhibited by acalabrutinib in a dose-dependent manner. Chemokine analysis from serum samples revealed significant differences in CXCL10, CXCL13, and CCL22 levels compared to the allogeneic control group. Furthermore, the acalabrutinib treatment showed decreased B220^+^ cells and CD3 infiltration in the skin. RNA-seq analysis of skin identified genes associated with keratinization that were downregulated with acalabrutinib treatment.

**Conclusion:**

Our results demonstrate that specific inhibition of BTK with acalabrutinib has a potentially beneficial role in ameliorating sclerodermatous cGVHD by improving the survival and clinical benefits and warrants further investigation in patients with cGVHD.

## 1 Introduction

Allogeneic hematopoietic stem cell transplantation (allo-HSCT) is a beneficial treatment option for patients with hematopoietic disorders. The advantageous outcomes are limited by the complication of graft-versus-host disease (GVHD) that occurs after allo-HSCT in up to 50% of patients, with high mortality and morbidity (1). The onset of GVHD can be classified into acute GVHD (aGVHD) and chronic GVHD (cGVHD), where aGVHD typically occurs within the first few months after allogeneic transplantation, affecting major organs such as skin, gastrointestinal tract, and liver (2). The pathophysiology of cGVHD is complex and not completely understood, and it is a major cause of morbidity and mortality with cumulative incidences ranging from 20-77%. cGVHD not only presents more like a chronic disease but also affects overall patient survival (3). Initially, cGVHD is thought of as T cell-mediated, but growing evidence suggests the role of B cells in the development and progression of the disease (4, 5). In this context, targeting B-cell-mediated therapies has been shown to be beneficial for preventing and treating cGVHD.

Bruton’s tyrosine kinase (BTK), a member of the TEC kinase family required for B cell receptor signaling (BCR) signaling, plays a major role in B cell survival, proliferation, development, and differentiation (2). Activation of BCR signaling involves BTK, which is crucial for the phosphorylation and activation of downstream effectors required for the development of cGVHD (2, 6). Given the beneficial effects of tyrosine kinase inhibitors, ibrutinib, a covalent inhibitor targeting BTK and ITK, has been approved by the FDA for the treatment of cGVHD (6). However, due to the off-target toxicities of ibrutinib, developing a second-generation and highly specific BTK inhibitor might contribute to improved benefits. Acalabrutinib irreversibly binds to Cys481, exhibiting improved selectivity and target engagement compared to ibrutinib in chronic lymphocytic leukemia patients. *In vitro* studies on primary human CLL cells showed acalabrutinib at greater selectivity for BTK across cysteine-containing kinases, and unlike ibrutinib did not inhibit EGFR, ITK, or TEC (7–10). BTK is an essential component of FcϵRI signaling in mast cells, which initiates a signaling cascade that involves mast cell activation and degranulation associated with cGVHD. We previously reported that mast cells are associated with fibrosis, which can be targetable for preventive and therapeutic interventions in cGVHD model, which, *in vitro*, were a target for ibrutinib as well (11).

Here, we hypothesize to examine the effects of Acalabrutinib, a second-generation BTK inhibitor for treating cGHVD in murine models after allo-HSCT. In this study, we used a well-established mouse model for sclerodermatous cGVHD after allo-HSCT to determine whether specific BTK inhibition using acalabrutinib is beneficial in reducing fibrosis and mast cells effector functions for the treatment of cGVHD.

## 2 Materials and methods

### 2.1 Animals

Animal strains BALB/cJ and B10.D2 mice were purchased from Jackson Laboratories, Bar Harbor, ME, USA, and approved by the University of Kentucky Institutional Animal Care and Use Committee and the Department of Laboratory Animal Resources. In this study, female mice of 9–12 weeks of age were used for transplant experiments. BTK inhibitor acalabrutinib was provided by AstraZeneca.

### 2.2 *In-vitro* study

Mast cells were generated ex vivo from bone marrow cells of BALB/cJ strain and cultured in DMEM media (12-604F, Lonza, Basel, Switzerland) with 10% heat-inactivated fetal bovine serum (35-011-CV, Corning Inc., Corning, NY) and 1% penicillin/streptomycin (120-095-721EA, Quality Biological, Gaithersburg, MD) at 37 °C with 5% CO2, in a humidified incubator. The suspension cells were transferred to a new flask after 24h in the presence of 10 ng/ml recombinant murine IL-3 (213-13, Peprotech, Rocky Hill, NJ) and passaged twice every week. The phenotype of the mast cells was confirmed by flow cytometry after 4–6 weeks. For the mast cell stimulation experiments, cells were incubated overnight with 100 ng/ml murine monoclonal anti-DNP IgE, clone SPE-7 (D8406, RRID: AB259249, Millipore-Sigma, St. Louis, MO) and washed twice to remove unbound IgE. Then, mast cells were stimulated by adding 10 ng/ml IL-33 and/or 100 ng/ml DNP-BSA along with drug treatments (acalabrutinib and ibrutinib or vehicle control) for 24 h at 37 °C, 5% CO2, in a humidified incubator. The cell supernatant was collected by centrifugation and stored at -80 °C for further analysis.

### 2.3 Transplant experiments

The sclerodermatous cGVHD murine model consisted of allo-HSCT across minor mismatches using B10.D2 to BALB/cJ. Following conditioning with total body irradiation (800cGy) of split dose using a cesium irradiator, recipients BALB/cJ received an infusion of 8 million bone marrow and 25 million splenocytes of either syngeneic (BALB/cJ) or allogeneic (B10.D2) hematopoietic cells. The mice were subsequently treated orally using oral gavage with acalabrutinib 25 mg/kg or placebo from week 3 to week 8. Mice were monitored for survival, scored weekly for clinical symptomology of GVHD, and euthanized on week 8 for analysis. Organs, including skin, lung, liver, colon, and small intestine, were collected in formaldehyde and transferred to 70% ethanol. Tissues were processed, embedded in paraffin blocks, and sectioned at 5-micron thickness for further analysis.

### 2.4 GVHD scoring

Recipient BALB/cJ mice were monitored daily for survival, and clinical GVHD was evaluated weekly in a blinded manner using a composite clinical scoring system based on five parameters as described (12). The severity and progression of GVHD were calculated using the clinical score index, in which each mouse received a score of 0 to 2 for each parameter, with a maximum score of 10.

### 2.5 Histology and analysis

Hematoxylin and eosin (H&E) stains were performed at the University of Kentucky Surgical Pathology core and assessed by a pathologist in a blinded fashion. Masson’s trichrome staining was performed to assess the dermal thickness of the tissues according to the manufacturer’s protocol (26367-Series, Electron Microscopy Sciences, Hatfield, PA). Trichrome slides were imaged using Evos M7000 microscopy (Thermoscientific, USA), and dermal thickness was measured using the ruler tool across 10–20 regions within the skin tissue images. Toluidine blue and Texas red staining were performed based on our previous reports (11). Images were captured using an Evos M7000 (Thermofisher Scientific, USA), and the number of positive cells was counted per high-power field.

### 2.6 Immunohistochemistry and immunofluorescence staining

The formalin-fixed paraffin sections were deparaffinized by incubation overnight at 60 °C in an oven, followed by dehydration in xylene, ethanol series (100%, 90%, 70%, 50%, and 30%), and subjected to antigen retrieval using citrate buffer. After retrieval, sections were washed in phosphate-buffered saline (PBS) and were blocked for 1h in goat serum, followed by incubation with respective primary antibodies for B220 (14-0452-82, Thermo Fisher Scientific, Waltham, MA) and CD3 (99940S, Cell Signaling Technology). After incubation with the primary antibody, slides were washed in PBST, followed by incubation with a secondary antibody depending on the primary antibody used. The sections were developed using a DAB substrate (Dako, Carpinteria, CA), counterstained with hematoxylin, and mounted with aqueous media. For Immunofluorescence staining, tissue sections were permeabilized and blocked (0.2% Triton X-100 in PBST, 1% BSA, and 10% normal horse serum) for 1 h at room temperature (RT). Then, slides were incubated overnight at 4 °C with Pan Cytokeratin Monoclonal Antibody (53-9003-82, Thermo Fisher Scientific, USA) and mounted using a Vectashield Hard Set Antifade Mounting Medium with DAPI (H-1500-10; Vector Laboratories). Images were captured using an Evos M7000 (Thermofisher Scientific, USA), and data were analyzed using ImageJ software (National Institutes of Health, Bethesda, MD, USA).

### 2.7 Measurement of cytokines and chemokines

The levels of TNF-α, IL-6, IFN-γ, IL-2, IL-4, IL-10, and IL-17α were determined in serum or cell supernatants by Mouse LEGENDplex MU Th17 Panel (741047, Biolegend) according to the manufacturer’s protocol. Measurement of chemokines in serum and supernatants from mast cell stimulation assays was analyzed with the LEGENDplex Mouse Proinflammatory Chemokine Panel (740007, Biolegend) per the manufacturer’s instructions, and values were normalized to the control. The samples were acquired using BD Biosciences LSRII flow cytometer (BD Biosciences) and analyzed using Biolegend LEGENDplex data analysis software.

### 2.8 RNA sequencing analysis

Whole skin from mice was used for total RNA extraction by RNeasy mini kit (74104, Qiagen) according to the manufacturer’s instructions. RNA concentration and quality were detected by Agilent 5200 fragment analyzer (Agilent Technologies, Inc., Santa Clara, CA, USA). Samples with RNA integrity more than 6.0 were used to construct RNA-Seq libraries using a poly A enrichment method and sequencing was performed using an Illumina NovaSeq 6000 platform. The quality assessment of initial raw RNA-Seq reads was executed through FastQC (Version 0.11.9), with subsequent consolidation of outcomes via the MultiQC tool (Version 1.11). The reads aligned to the reference Mus musculus (house mouse) genome (GRCm39.107) using HISAT2 (Version 2.2.1), resulting in a robust overall alignment rate of about ~95%. Further processing involved the conversion of the aligned files into sorted BAM files using Sam tools (Version 1.14). Notably, Cufflinks (Version 2.2.1) played a key role in generating gene expression abundance levels, which in turn furnished FPKM values. These FPKM values were pivotal for constructing PCA plots, enabling a comprehensive assessment of intrinsic differences among the samples. In our study, we employ Cufflink’s Cuffdiff tool to investigate the gene responses within a dataset comprising 16 samples. We conduct 4 distinct comparisons (AT_vs_AC, ST_vs_SC, AC_vs_SC, and AT_vs_ST), SC- syngeneic control, ST- syngeneic acalabrutinib treated, AC- Allogeneic control, AT- Acalabrutinib treated as meticulously examining alterations triggered by treating samples with Syn and Allogeneic with Control and Acalabrutinib groups. Our analysis homes in on genes displaying a minimum twofold change ensuring the robustness of our findings (supported by a q-value of <0.05). These stringent criteria facilitate the identification of differentially expressed genes (DEGs), providing insights into unique treatment responses. This meticulous approach enables us to precisely pinpoint the pivotal distinctions between treatments. The Cluster Profiler R package has been used to perform KEGG and GO enrichment analysis and to plot the results as dot plots.

### 2.9 Statistical analysis

Statistical analysis was done by the two-tailed independent t-test, one-way ANOVA, with a Tukey’s *post-hoc* multiple comparisons test, wherever appropriate, with P values ≤0.05 considered statistically significant. P value 0.01 to 0.05 = *, P value 0.001 to 0.01 = **, P value 0.0001 to 0.001 = ***, P value <0.0001 = ****, NS = not significant. All figures were prepared in GraphPad Prism version 9.0 software package (GraphPad, CA, USA).

## 3 Results

### 3.1 Acalabrutinib treatment improves survival, clinical outcomes, and reduces sclerodermatous cGVHD

**Figure 1:**
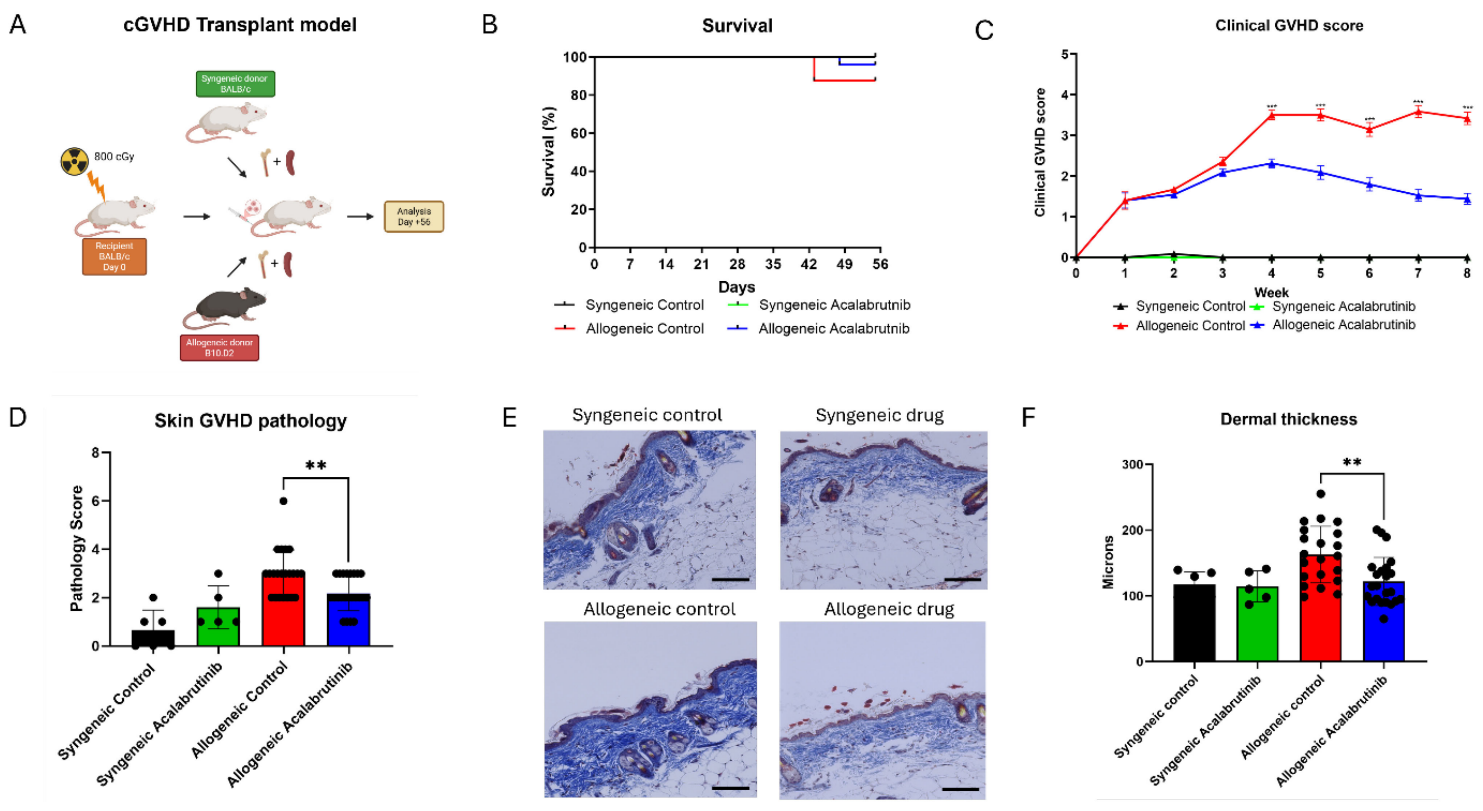
Acalabrutinib improves clinical outcomes and decreases sclerodermatous cGVHD after allogeneic HSCT **(A)** Schematic representation of cGVHD transplant model **(B)** Kaplan-Meier survival curve of syngeneic (n = 6), syn-acalabrutinib (n = 5), allogeneic (n = 21), allo- acalabrutinib (n = 23) groups after transplant. **(C)** Weekly GVHD scoring of groups after transplant. Error bars are the standard error of the mean (SEM). Clinical GVHD scores were calculated based on parameters including posture, mobility, skin, fur and weight loss **(D)** Skin pathology scores and **(E)** Representative images and quantification of Masson’s trichrome staining of skin from syngeneic (n = 6), syn-acalabrutinib (n = 5), allogeneic (n = 21), allo- acalabrutinib (n = 23) groups after transplant. Scale bar: 150 μm. **(F)** Quantification is the combination of data from two independent transplants. **P = 0.001–0.01, ***P = 0.0001–0.001.

In this study, we aimed to investigate the effects of acalabrutinib on sclerodermatous cGVHD utilizing our established cGVHD model depicted in **Figure 1A**. Syngeneic and allogeneic groups were treated with acalabrutinib or vehicle control and analyzed on day +56. Syngeneic groups served as a negative control that would not develop GVHD, and no deaths were observed, demonstrating tolerability and safety in the acalabrutinib syngeneic control group. Treatment with acalabrutinib in the allogeneic group showed better survival than in allogeneic controls (**Figure 1B**). The allogeneic group demonstrated a significant increase in GVHD score, depicting the progression of GVHD and a reduced survival rate. Treatment with acalabrutinib significantly reduced clinical GVHD scores and correlated with the survival benefits (**Figure 1C**). In this study, the B10.D2 to BALB/cJ model prominently displayed skin’s sclerodermatous cGVHD, which allowed us to assess the effect of acalabrutinib on skin fibrosis and inflammation. H&E staining results showed that the allogeneic control group has the highest pathology as scored by a pathologist in a blind fashion, demonstrating skin fibrosis and inflammation, which validates our sclerodermatous model. Treatment with the BTK inhibitor of the allogeneic group significantly reduced the skin pathology scores compared to the allogeneic control group (*P* = 0.002) (**Figure 1D**). There was no significant difference observed in the organ’s (lung, liver, small intestine, and colon) pathology compared to allogeneic controls (data not shown). The presence of dermal thickness as a characteristic of sclerodermatous cGVHD was assessed by Masson’s trichrome staining. A significant increase in dermal thickness was evident in the allogeneic control group, measured by the presence of a blue collagenous layer in the dermis compared to the syngeneic control group (**Figures 1E, F**). Treatment with acalabrutinib significantly reduced the dermal thickness in allogeneic recipients (*P* = 0.001) as evidenced by a thin blue collagenous layer, which displayed the effect of BTK on mitigating fibrosis.

### 3.2 Immune cell infiltration of the skin is ameliorated by acalabrutinib

**Figure 2:**
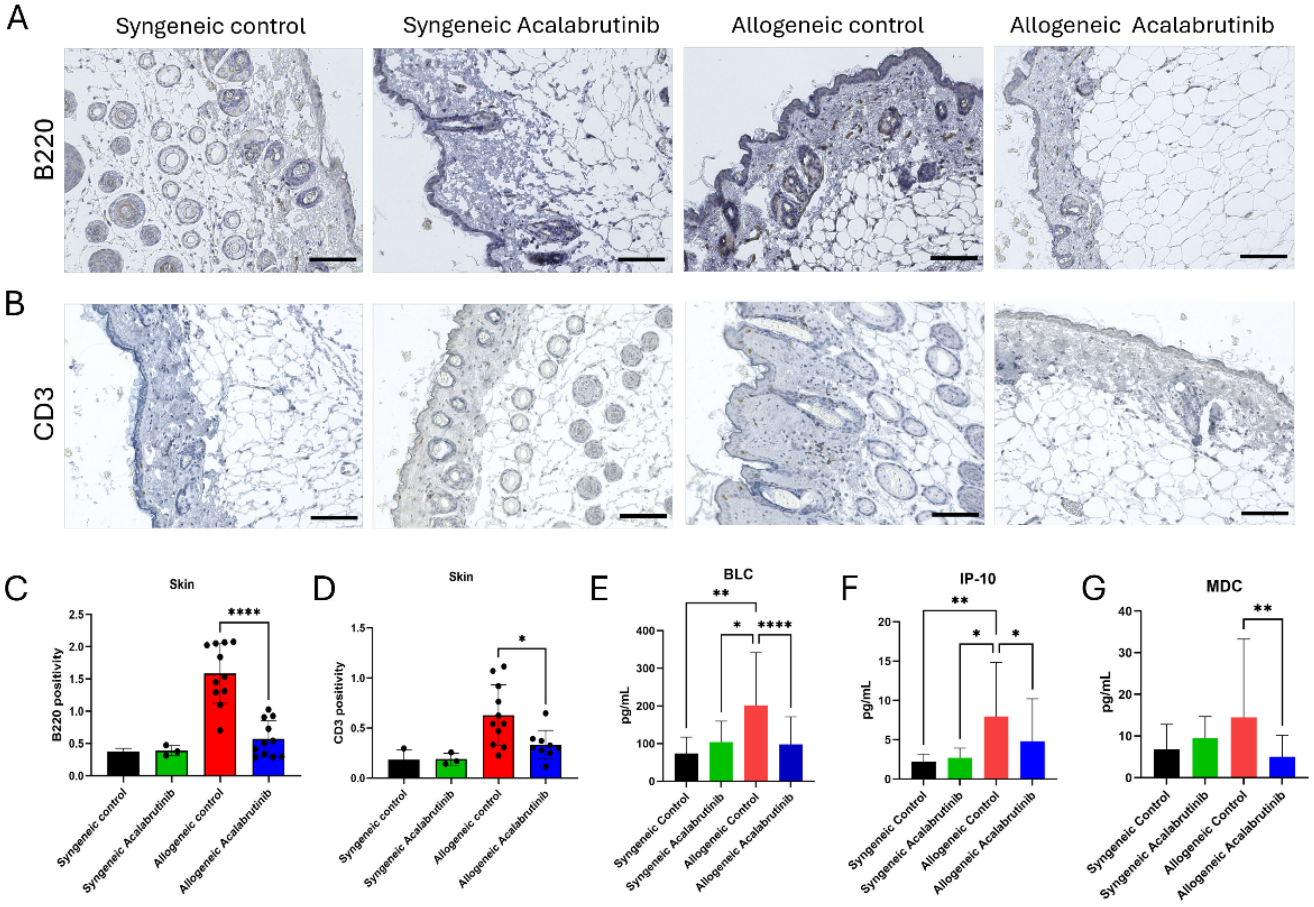
Immune infiltration and chemokine production are ameliorated by acalabrutinib treatment **(A)** Serum chemokine assays were analyzed after transplant using the LEGENDplex Inflammatory Chemokine Assay kit. **(B, C)** Representative images and quantification of B220^+^ staining of skin after transplant. **(D, E)** Representative images and quantification of CD3 staining of skin after transplant. Scale bar: 150 μm. Data were expressed as mean ± SD. *P = 0.01–0.05, **P = 0.001–0.01, ****P < 0.0001.

The involvement of CD4^+^ T cells and B cells significantly mediates the pathogenesis of cGVHD via activation, antigen-presentations, and antibody-production, causing tissue damage and fibrosis (13). Given the inhibitory effect of acalabrutinib on BCR signaling, we aimed to determine whether BTK inhibition is beneficial in reducing inflammation and chemokine signaling in our model of sclerodermatous skin GVHD. Serum chemokine analysis showed significantly reduced levels of CXCL13, CXCL10, and CCL22 in allogeneic acalabrutinib treated groups (**Figures 2E–G**). These chemokines can be chemotactic for the recruitment of B lymphocytes, T-cells, and other effector cells that are implicated in cGVHD pathogenesis (14, 15). Further, we determined immune cell infiltration by immunohistochemistry analysis, which showed significantly reduced B220^+^ B cell infiltration in the skin of allogeneic acalabrutinib treated recipients (**Figures 2A, C**). These results suggest that acalabrutinib treatment reduced CXCL13, signaling chemotactic for B cells to the target sites of inflammation associated with reduced B cell infiltration. Interestingly, allogeneic recipients treated with acalabrutinib also demonstrated significantly reduced T-cell infiltration in the skin (**Figures 2B, D**), representing that cascade of T- and B-cell priming is essential for cGVHD pathogenesis.

### 3.3 Acalabrutinib mitigates sclerodermatous cGVHD via targeting mast cells

**Figure 3:**
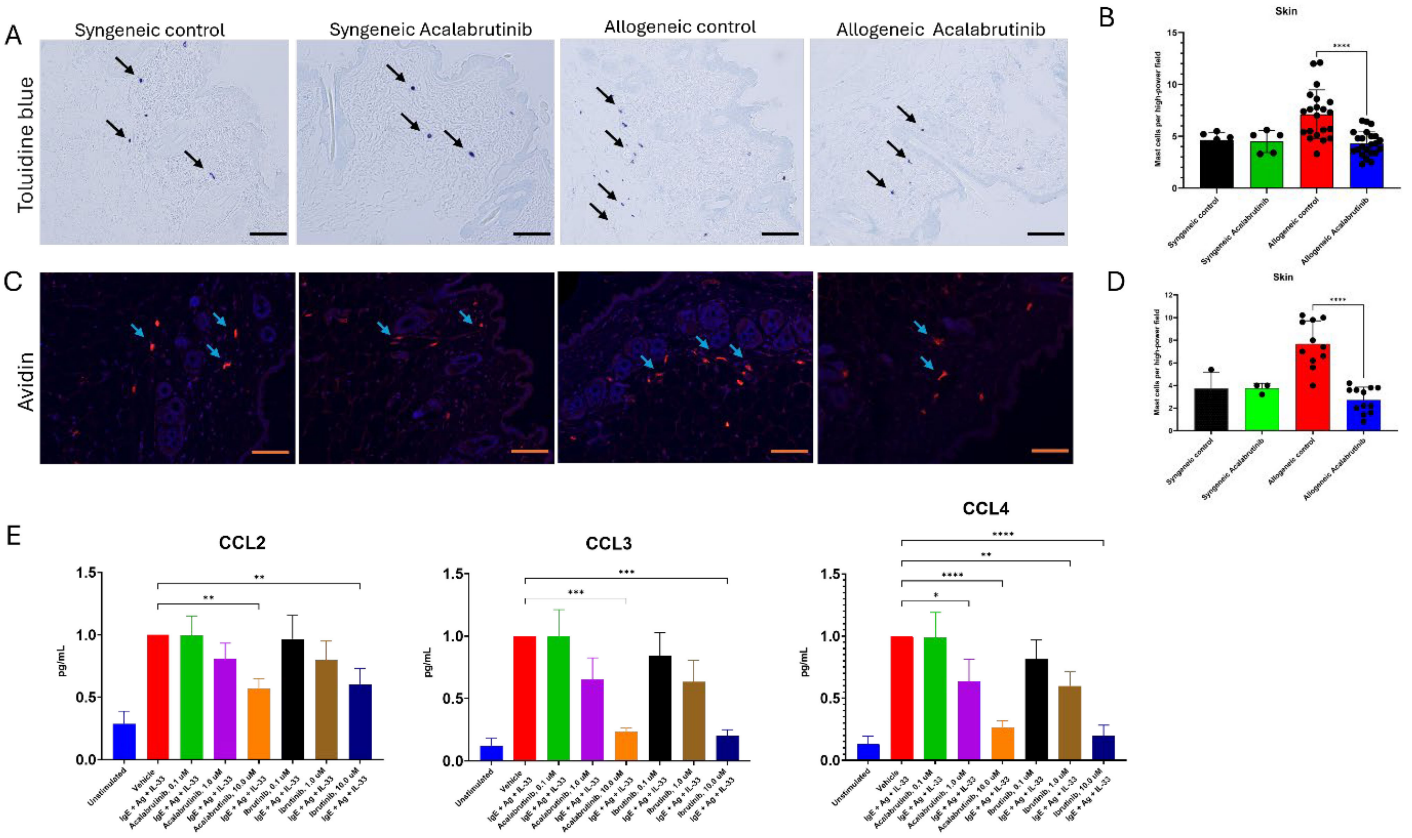
Mast cell infiltration in the dermal environment is mitigated by acalabrutinib treatment. **(A, B)** Representative images and quantification of toluidine blue-stained skin and mast cell counts of skin. **(C, D)** Representative images from avidin-stained skin (blue = DAPI, red = Avidin) and mast cell counts from the cGVHD model. Scale bar: 150 μm. **(E)** Mast cell mediated production of chemokines is inhibited by acalabrutinib. Hematopoietic cells were cultured with IL-3 for differentiation and sensitized with IgE for 24 h. The cells were stimulated with IL-33 for 24 h with inhibitors, collected supernatant and analyzed using the LEGENDplex inflammatory chemokine assay kit. Data were expressed as mean ± SD. *P = 0.01–0.05, **P = 0.001–0.01, ***P = 0.0001–0.001, ****P < 0.0001.

Our previous studies have demonstrated that mast cells are critical mediators of fibrosis in cGVHD models of the skin (11). In this current study, we were interested in examining the effects of BTKi on mast cells *in vitro* and *in vivo*. Seven weeks after transplant, we used histochemical methods to analyze mast cells in the skin tissues. Toluidine blue staining revealed a low number of mast cells in the syngeneic and allogeneic acalabrutinib treated groups compared to the allogeneic control group (**Figures 3A, B**). In addition to confirming these findings obtained from toluidine blue staining, avidin staining was performed, conjugated to Texas red fluorophore stains of mast cells in a biotin-dependent manner. Avidin staining of skin tissues showed a similar correlation to that of toluidine staining, where a significant reduction in the mast cell numbers in the allogeneic acalabrutinib treated group compared to the allogeneic control group was observed (**Figures 3C, D**).

### 3.4 BTK inhibition suppressed mast cell-mediated chemokine production

We previously reported that mast cell-mediated chemokine production is critical in the pathogenesis of sclerodermatous GVHD (11). Here, we want to examine whether mast cells produced chemokines upon stimulation with IgE/antigen crosslinking or IgE/antigen + IL-33 were inhibited by acalabrutinib. *In vitro*, stimulation of murine mast cells demonstrated increased pro-inflammatory chemokines (CCL2, CCL3, and CCL4) that were effectively inhibited by both acalabrutinib and ibrutinib in a dose-dependent manner (**Figure 3E**). The over-expression of these chemokines in the skin of allogeneic recipients was associated with fibrosis development (11). The inhibition of this mast cell chemokine production by acalabrutinib was comparable to Ibrutinib, which is a clinically efficacious drug in treating cGVHD patients.

### 3.5 Transcriptomics analysis of cGVHD skin

**Figure 4:**
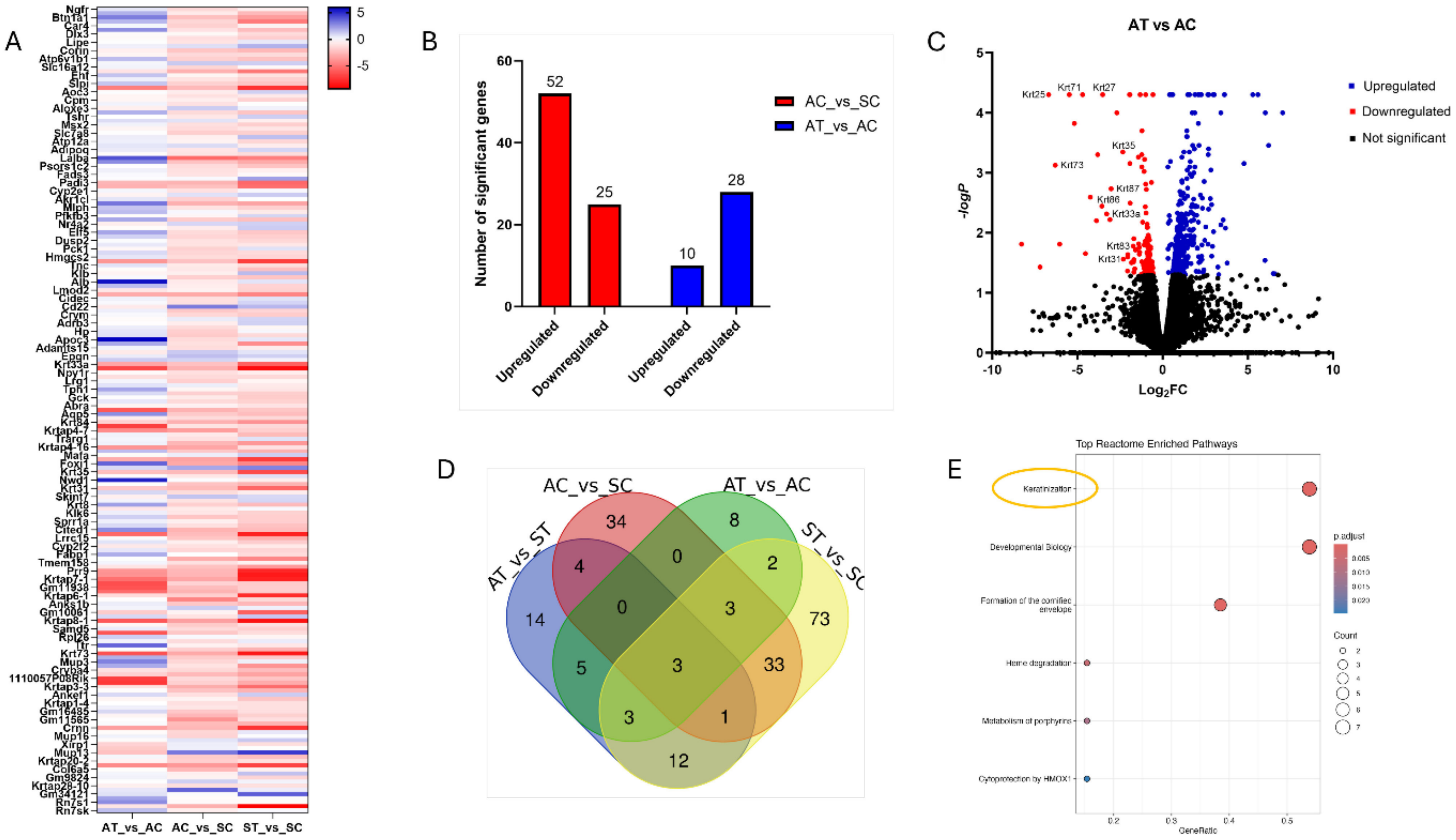
Transcriptomic analysis of cGVHD by RNA-seq analysis of skin samples after transplant. **(A)** Heat map of gene expression profiles in different groups. **(B)** Differential gene counts between different groups. **(C)** Volcano plots. **(D)** Venn diagram of differential gene counts. **(E)** Pathway analysis based on DEG’s from different groups.

**Figure 5:**
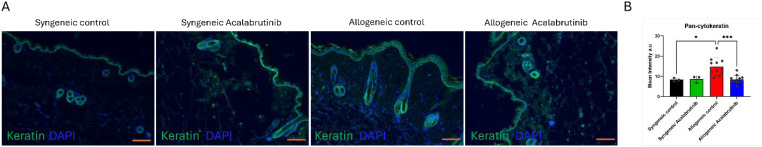
Cytokeratin expression in cGVHD skin is reduced by acalabrutinib treatment **(A, B)** Representative images and quantification of pan-cytokeratin staining of skin after transplant. Scale bar: 150 μm. Data were expressed as mean ± SD. *P = 0.01–0.05, ***P = 0.0001–0.001, ****P < 0.0001.

RNA sequencing was performed to identify the regulatory genes associated with acalabrutinib treatment in the skin of GVHD mice. Our results revealed that differentially expressed genes among the groups were demonstrated by a heatmap and as well as differentially expressed gene counts (DEG) (**Figures 4A, B**). In total, 195 DEGs were observed among the groups (**Figure 4A**), whereas only limited DEGs (10 upregulated, 28 downregulated) were recorded in the acalabrutinib allo-treated vs allo-control group (**Figure 4B**). The analysis of the chosen DEGs by Reactome aimed to identify the primary regulatory signaling pathway in mice treated with acalabrutinib. The top signaling pathways were listed based on the -log (p-value) score, and among them, the keratinization pathway was impacted the most by acalabrutinib treatment, with downregulated genes including Krt25​, Krt27, Krt71​, Krt73 (**Figures 4C, E**). The downregulated keratins are hair follicle-specific epithelial keratins type I (Krt25, Krt27) and type II (Krt71, Krt73), specifically expressed in hair follicle inner root sheath (IRS) (16, 17). Next, we want to determine the expression of cytokeratins in the skin of allogeneic recipients. Immunofluorescence analysis of pan-cytokeratin (a marker of type I and type II keratins) in the skin showed over-expression of cytokeratin in allogeneic control-treated recipients in the epidermal and IRS regions and was significantly reduced in the allogeneic acalabrtuinib treated group, with an expression level comparable to the syngeneic group (**Figures 5A, B**).

## 4 Discussion

The pathophysiology of cGVHD is complex and not well understood, resulting in the lack of effective therapies. This, despite recent advances in the management of cGVHD, remains an unmet need and a major cause of morbidity and mortality after allo-HSCT (3). In cGVHD, cutaneous manifestations lead to alterations in essential barrier functions and infections, reflecting a spectrum of epidermal and dermal changes (18). cGVHD development involves both B and T cells. Cell functions are regulated in part by TEC kinases, including BTK and ITK. In recent years, growing evidence in animal and human studies has demonstrated the prominent role of B cells in the development of cGVHD, other than T-cells (3). It has been shown that recipients given splenocytes with ITK deficiency or lacking functional BTK do not develop cGVHD after allo-BMT, indicating the importance of BTK or ITK in cGVHD development (6). Several studies reported that BTK and ITK are potential therapeutic targets that can be inhibited by Ibrutinib and Tec kinase inhibitors and displayed beneficial effects in several acute and chronic GVHD preclinical models (6, 13, 19). Moreover, BTK is also expressed in other cell types of the hematopoietic lineage including dendritic cells (DCs), mast cells, and macrophages suggesting the inhibition of BTK and its downstream effectors can be beneficial in treating cGVHD leveraging other pathways beyond BCR signaling in B cells. Here, we show that BTK inhibition with second generation inhibitor Acalabrutinib reduced the pathogenesis and clinical manifestations in our murine model of sclerodermatous GVHD by affecting mast cells.

cGVHD can affect many organs and systems and the underlying mechanisms include allo-antigen specific donor T cell activation, loss of regulatory cell populations, disruption to B cell homeostasis and fibrosis (18, 20) Although skin is the most affected site in cGVHD very few studies have focused on understanding the mechanisms of pathogenic fibrosis in cGVHD. The major hallmark of sclerodermatous cGVHD is characterized by skin fibrosis associated with thickening of collagen bundles throughout the dermal regions. During the progression of cGVHD, activated macrophages produce TGF-β, which drives the differentiation of fibroblasts into myofibroblasts that promote collagen deposition in the skin (18). In our study, we found that inhibition of BTK significantly reduces the dermal collagen deposition that could be associated with mast cell-mediated proliferation of fibroblasts. Earlier, we reported that mast cells act as mediators of fibrosis and effector cell recruitment in this sclerodermatous cGVHD model. Mast cells are one of the key sources of fibroblast growth factor that induces proliferation in fibroblasts and leads to collagen deposition, and mast cell-deficient mice showed a reduction in skin pathology, dermal thickness, and fibrosis after allo-transplant (11) It has been shown, that murine mast cells with genetic BTK deficiency showed reduced production of proinflammatory cytokines such as IL-12, TNF-α, and IL-6 revealing the involvement of BTK in mast cells in GVHD (21). In this study, acalabrutinib treatment reduces mast cell numbers and mast cell-mediated chemokine production, suggesting that BTK inhibition by acalabrutinib primarily affects mast cell functions along with B cells, which could prevent or delay the onset of disease. Mast cell activation by cytokinergic IgE may be generated by local germinal center reactions in the target organs, which could initiate a positive feedback loop mechanism between mast cells and B cells that induces the production of cytokines (22).

The production of autoantibodies by donor B cells within the germinal center of lymphoid organs initiates tissue inflammation and GVHD development. In cGVHD, B cells also function as antigen presenting cells that induce the expansion of pathogenic CD4^+^ T cells (23). We found that acalabrutinib was able to reduce the B cells expression in skin of allo-recipients suggesting the efficacy of BTK inhibition. These results were correlated with previous findings demonstrating that Ibrutinib, a specific BTK/ITK inhibitor reduces pulmonary B cell infiltration in cGVHD murine model (6), both showing decreased cGVHD development cGVHD pathogenesis.

The spatial and temporal transcriptional signatures associated with cutaneous GVHD development related to inflammation, apoptosis, effector cell recruitment and activation and tissue repair in rodent models associated with *in situ* morphological alterations (24, 25). However, correlating the available knowledge into a framework describing the cutaneous GVHD progression and identifying targetable pathways remains challenging. In our study, we found that impaired control of keratin expression in the epidermis and inner root sheath in the allogeneic recipients along with a significant increase in epidermal thickness might be due to dysregulated epithelial cell proliferation and altered terminal differentiation. Increasing evidence supports the epidermis’s major role in promoting dermal fibrosis (26–28). Looking at epithelial cells during tissue repair, activated keratinocytes in the epidermis promote fibroblast activation and collagen deposition leading to fibrosis in systemic sclerosis (29, 30). In dermal chronic GVHD samples, the epidermis showed elevated expressions of KT6A/B, K16, K17 levels which can lead to hyperproliferation, aberrant innate immunity activation and delayed wound healing. Inflammatory keratinocyte reprograming promotes feed-forward loop of enhanced inflammation, leukocyte infiltration and activation in cutaneous GVHD (31). Acalabrutinib reduced the elevated keratin expression in allogeneic recipients, suggesting that it may interrupt keratinocyte-fibroblast crosstalk, potentially through mast-cell mediated pro-fibrotic signaling which needs deeper understanding and allows us to develop future research directions in sclerodermatous cGVHD. In summary, our study demonstrates that specific inhibition of BTK with second generation inhibitor acalabrutinib reduces cGVHD of the skin via its effects on mast cells, B cells and tissue stroma. Further clinical studies are warranted to explore the potential of this selective BTK inhibitor in the management of clinical cGVHD.

## Data Availability

The datasets presented in this study can be found in online repositories. The names of the repository/repositories and accession number(s) can be found below: https://www.ncbi.nlm.nih.gov/, GSE310029.
